# Characterising the evolution of antipsychotic polypharmacy and clozapine prescribing patterns in schizophrenia patients during psychiatric hospitalisations

**DOI:** 10.1192/j.eurpsy.2022.284

**Published:** 2022-09-01

**Authors:** J. Lagreula, L. Elens, P. De Timary, O. Dalleur

**Affiliations:** 1 Université Catholique de Louvain, Louvain Drug Research Institute (LDRI), Clinical Pharmacy Research Group (clip), Brussels, Belgium; 2 Université Catholique de Louvain, Louvain Drug Research Institute, Integrated Pharmacometrics, Pharmacogenomics And Pharmacokinetics, Brussels, Belgium; 3 Université Catholique de Louvain, Institute Of Neuroscience, Brussels, Belgium; 4 Université Catholique de Louvain, Louvain Drug Research Institute, Clinical Pharmacy Research Group, Brussels, Belgium

**Keywords:** clozapine, Psychiatric hospitalisations, Antipsychotic polypharmacy, Clinical pharmacy

## Abstract

**Introduction:**

A high prevalence of antipsychotic polypharmacy (APP) and low utilisation of clozapine is considered as inappropriate prescribing that can lead to suboptimal treatment, increased risk of poor response or adverse effects.

**Objectives:**

To explore the evolution of prevalence of APP and associated factors as well as clozapine prescribing patterns between hospital admission and discharge.

**Methods:**

We collected retrospective data on adult inpatients diagnosed with schizophrenia spectrum disorders in 2020-2021 in 6 Belgian hospitals.

**Results:**

Of the 516 patients analysed, APP prescribing significantly increased from 47.9% on hospital admission to 59.1% at discharge. Both on admission and at discharge, APP was associated with treatment with a first-generation antipsychotic, not being treated with an antidepressant nor a mood stabilizer, high antipsychotic dosage, increased number of psychoactive cotreatments and total medicines. A lower number of comorbidities (OR=0.68, CI=0.50-0.91), no treatment with benzodiazepines (OR=0.02, CI=0.01-0.09) nor with trazodone or sedative antihistamines (OR=0.06, CI=0.01-0.03) and two or more previous antipsychotic trials (OR=4.91, CI=1.30-18.57) was associated with APP on admission only. APP at discharge was more frequent in patients with antipsychotic adverse effects (OR=2.57, CI=1.10-6.00), prior clozapine use (OR=16.30, CI=3.27-81.22) and not involuntary admitted (OR=0.26 CI=0.08-0.88). Contrary to admission, treatment with benzodiazepines was associated with APP at discharge (OR=10.9, CI=3.38-5.38). Only 9.3% of admitted patients were treated with clozapine. Although 28.1% were eligible, clozapine was introduced to 10 patients leading to 11% being discharged on it.

**Conclusions:**

Inappropriate prescribing of antipsychotics to schizophrenia patients persist after psychiatric hospitalisations and are associated with identifiable characteristics.

**Disclosure:**

No significant relationships.

